# RETRACTION: “Preparation and Characterization of Polyhydroxybutyrate/Polycaprolactone Nanocomposites”

**DOI:** 10.1155/tswj/9897271

**Published:** 2025-08-20

**Authors:** The Scientific World Journal

RETRACTION: C.P. Liau, M.B. Ahmad, K. Shameli et. al., “Preparation and Characterization of Polyhydroxybutyrate/Polycaprolactone Nanocomposites,” *The Scientific World Journal* 2014 (2014): 572726, https://doi.org/10.1155/2014/572726.

The above article, published online on 29 January 2014 in Wiley Online Library (wileyonlinelibrary.com), has been retracted by John Wiley & Sons Ltd and the journal's Associate Editor; Claudia Masselli.

The retraction has been agreed following an investigation into concerns raised by a third party, which identified unexpected similarity with another article published by the same author group.

More specifically, the TEM image in Figure 5b appears similar to Figure 9 in another publication [1], where it denotes a different nanocomposite material.

The authors did not respond to our request for clarification and as a result, the conclusions of the current article are considered unreliable.

The authors have been informed of the retraction but did not provide a response.
